# Decreased Excretion of Urinary Exosomal Aquaporin-2 in a Puromycin Aminonucleoside-Induced Nephrotic Syndrome Model

**DOI:** 10.3390/ijms21124288

**Published:** 2020-06-16

**Authors:** Ahmed Abdeen, Hiroko Sonoda, Ayaha Kaito, Sayaka Oshikawa-Hori, Naruki Fujimoto, Masahiro Ikeda

**Affiliations:** 1 Department of Veterinary Pharmacology, University of Miyazaki, Miyazaki 889-2192, Japan; ahmed.abdeen@fvtm.bu.edu.eg (A.A.); sonoda-h@cc.miyazaki-u.ac.jp (H.S.); a8888thomas@gmail.com (A.K.); oshikawa.sayaka.g3@cc.miyazaki-u.ac.jp (S.O.-H.); gf14024@student.miyazaki-u.ac.jp (N.F.); 2 Department of Forensic Medicine and Toxicology, Faculty of Veterinary Medicine, Benha University, Toukh 13736, Egypt

**Keywords:** urinary exosomes, aquaporin-2, puromycin aminonucleoside, nephrotic syndrome, nephrotoxicity

## Abstract

Urinary exosomes, small extracellular vesicles present in urine, are secreted from all types of renal epithelial cells. Aquaporin-2 (AQP2), a vasopressin-regulated water channel protein, is known to be selectively excreted into the urine through exosomes (UE-AQP2), and its renal expression is decreased in nephrotic syndrome. However, it is still unclear whether excretion of UE-AQP2 is altered in nephrotic syndrome. In this study, we examined the excretion of UE-AQP2 in an experimental rat model of nephrotic syndrome induced by the administration of puromycin aminonucleoside (PAN). Rats were assigned to two groups: a control group administered saline and a PAN group given a single intraperitoneal injection of PAN (125 mg/kg) at day 0. The experiment was continued for 8 days, and samples of urine, blood, and tissue were collected on days 2, 5, and 8. The blood and urine parameters revealed that PAN induced nephrotic syndrome on days 5 and 8, and decreases in the excretion of UE-AQP2 were detected on days 2 through 8 in the PAN group. Immunohistochemistry showed that the renal expression of AQP2 was decreased on days 5 and 8. The release of exosomal marker proteins into the urine through UEs was decreased on day 5 and increased on day 8. These data suggest that UE-AQP2 is decreased in PAN-induced nephrotic syndrome and that this reflects its renal expression in the marked proteinuria phase after PAN treatment.

## 1. Introduction

Urinary exosomes (UEs) are tiny extracellular vesicles (<100 nm) released from the apical membrane of the renal epithelium into the urinary space. Exosomes are known to be synthesized as intraluminal vesicles in multivesicular bodies (MVBs) in a process controlled by a set of cellular components including the endosomal sorting complex required for transport (ESCRT) machinery [1]. Thereafter, intraluminal vesicles are released extracellularly as exosomes upon fusion of the MVBs with the cell membrane [1,2]. Exosomes have been shown to carry membrane and cytosolic proteins, mRNAs, micro RNAs, long noncoding RNAs, and other molecules, and these molecule-laden exosomes are considered to play a role in intercellular communication under both physiological and pathological conditions [3,4,5,6]. Accumulative evidence has indicated that the relative abundance of these molecule-bearing urinary exosomes is altered in various kidney diseases [7]. In fact, it has been proposed that several exosome-derived molecules, such as aquaporins (AQPs) [8], miR-125a and miR-351 [6], RNA (MEG3) [9], RNA HOTAIR [4], and fetuin-A, could have potential application as biomarkers [10].

Aquaporin-2 (AQP2), a vasopressin-regulated water channel protein responsible for renal water reabsorption, is expressed in the principal cells of collecting ducts [11] and when present in the urine, it is known to be localized mainly in urinary exosomes (UE-AQP2) [12]. Our previous research has shown that UE-AQP2 is released into the urine at an early to late stage under renal pathological conditions such as treatment with gentamicin [13] or cisplatin [14], renal transplantation [15], and ischemia/reperfusion (I/R) [16].

The nephrotic syndrome is known to be associated with a urinary concentration defect in humans and experimental animal models [17,18,19]. Furthermore, in models of nephrotic syndrome induced by either PAN or adriamycin, decreased expression of renal AQP2 has been observed [18,19]. These findings strongly suggest that the excretion of UE-AQP2 is altered under the conditions of nephrotic syndrome, but this issue has not yet been investigated. In the present study, we investigated the excretion of UE-AQP2 in nephrotic syndrome using rats treated with puromycin aminonucleoside (PAN), as well as the release of exosomal marker proteins, including tumor susceptibility gene 101 protein (TSG101) and ALG-2 interacting protein X (ALIX) [8,20].

## 2. Results

### 2.1. Blood and Urine Parameters after PAN Treatment

Blood samples were obtained at the time of kidney removal on days 2, 5, and 8, and the results from blood biochemistry are shown in Table 1. In comparison with the control group, plasma creatinine levels were significantly increased on days 5 and 8 in the PAN group. On day 5, PAN caused hyponatremia and hyperchloremia. On day 8, alkalemia and hyponatremia were observed in the PAN group.

Urinalysis data are shown in Table 2. PAN caused significant decreases in urinary creatinine concentration and volume, accompanied by proteinuria on days 5 and 8, in comparison with the control group. Significant decreases in the excretion of urinary electrolytes including Na^+^, K^+^, and Cl^−^ and urinary acidification were also observed at all time points examined in the PAN group. These blood biochemistry and urinalysis data confirmed the successful induction of nephrotic syndrome.

### 2.2. Kidney Histology after PAN Treatment

**Figure 1:**
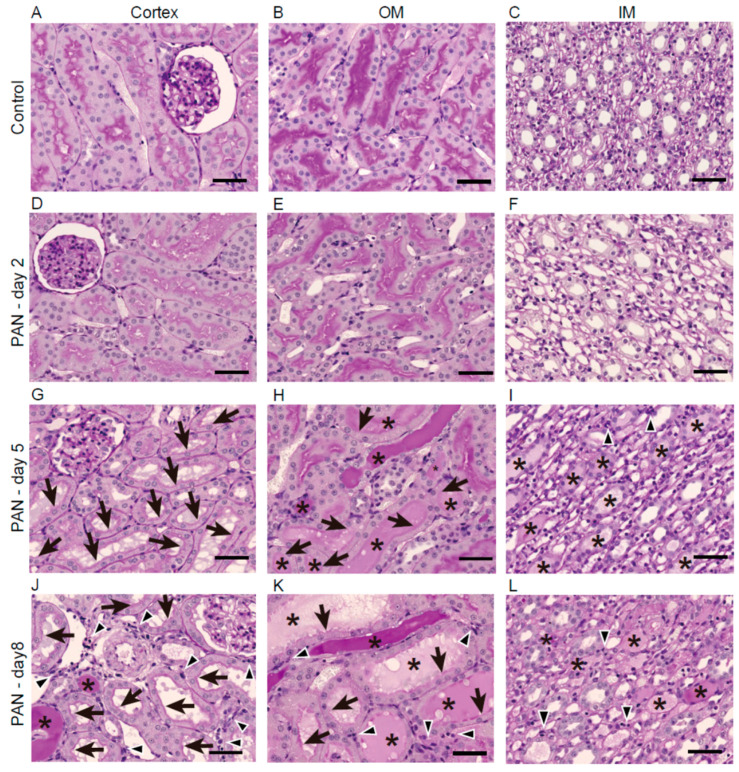
Renal histology after PAN treatment. (**A**–**L**) Representative images of kidney sections from the cortex, outer medulla (OM), and inner medulla (IM) of the control and puromycin groups (on days 2, 5, and 8) stained with periodic acid–Schiff reagent. Arrows, arrowheads, and * indicate loss of the brush border, interstitial cellular infiltration, and urinary casts, respectively. Bars = 50 µm.

As shown in Figure 1, histological analyses revealed renal tubular injury on days 5 and 8 (Figure 1G–L), but not on day 2 (Figure 1D–F). On day 5, kidney specimens obtained from the PAN group showed loss of the brush border, tubule necrosis, cast formation, moderate tubule dilatation, and interstitial lymphocytic infiltration in all regions of the kidney (Figure 1G–I). These kidney abnormalities were most sever on day 8 (Figure 1J–L).

### 2.3. Excretion of UE-AQP2 after PAN Treatment

**Figure 2:**
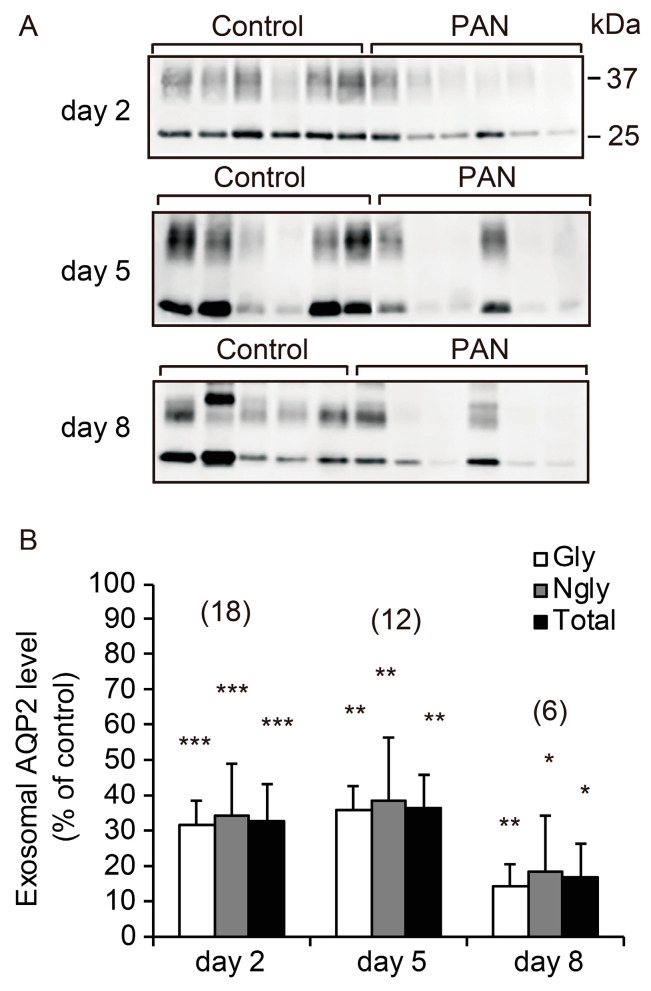
Urinary excretion of urinary exosomal aquaporin-2 (UE-AQP2) after PAN treatment. Representative immunoblots of UE-AQP2 (**A**) with quantitative data (**B**). Urine was collected for 6 h on days 2, 5, and 8. Each sample was loaded with the same amount of creatinine (80 µg/lane). Each value was calculated as a percentage of the mean value for the control group. Representative immunoblots show two bands: an upper band of glycosylated AQP2 (Gly, 37 kDa) and a lower band of non-glycosylated AQP2 (Ngly, 25 kDa). Data are expressed as means ± SE. * *p* < 0.05, ** *p* < 0.01, and *** *p* < 0.001 vs. control group.

We analyzed the excretion of UE-AQP2 after PAN treatment. As shown in Figure 2, PAN significantly decreased the excretion of UE-AQP2 at all time points examined relative to the control group.

### 2.4. Renal AQP2 Expression after PAN Treatment

**Figure 3:**
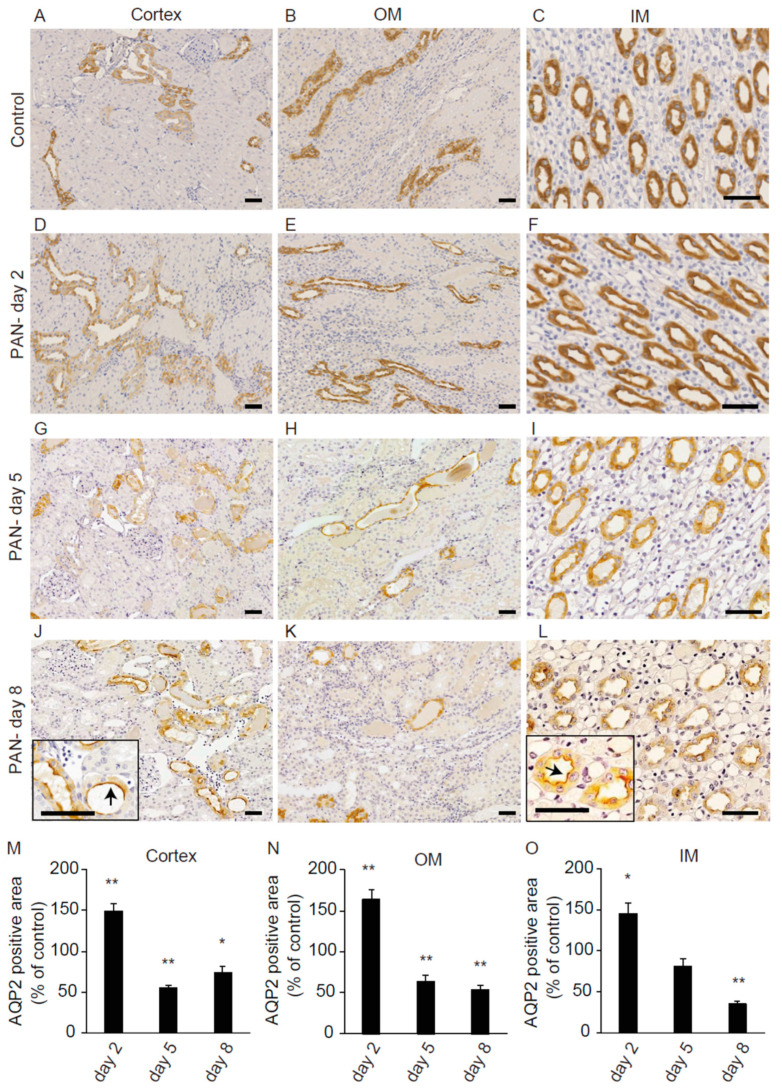
Immunohistochemistry of renal AQP2 after PAN treatment. Immunohistochemistry of renal AQP2 in the cortex (**A**,**D**,**G**,**J**), OM (**B**,**E**,**H**,**K**), and IM (**C**,**F**,**I**,**L**) after saline treatment on day 2 (**A**–**C**) or puromycin treatment on day 2 (**D**–**F**), day 5 (**G**–**I**), and day 8 (**J**–**L**). The black boxes in (**J**,**L**) indicate highly magnified renal tubules showing increased apical expression of AQP2. Bars = 50 µm. The AQP2-positive areas in the cortex (**M**), OM (**N**), and IM (**O**), in the PAN group relative to the control group are shown. Data are expressed as means ± SE. * *p* < 0.05 and ** *p* < 0.01 vs. the control group.

We next performed immunohistochemical analyses of renal AQP2 expression. Representative results of immunostaining are shown in Figure 3. PAN little affected the abundance of AQP2 on day 2. On day 5, there was an evident reduction in the expression of AQP2 in the cortical and corticomedullary layers, with less effect on the inner medulla (IM). On day 8, its expression was reduced in all kidney regions. Interestingly, we observed that apical expression of AQP2 was increased in some collecting duct cells on day 8, as shown in Figure 3J,L. The AQP2-positive area was measured semi-quantitatively (Figure 3M–O), and the mean ± SEM values in the PAN group relative to the control group were 149.8 ± 8.7% (*n* = 3, *p* < 0.01) in the cortex, 163.7 ± 12.5% (*p* < 0.01) in the outer medulla (OM), and 145.4 ± 13.1% (*p* < 0.05) in the IM on day 2, 55.2 ± 4.1% (*p* < 0.01) in the cortex, 63.9 ± 7.7% (*p* < 0.01) in the OM, and 81.5 ± 8.7% in the IM on day 5, and 73.8 ± 8.6% (*p* < 0.05) in the cortex, 53.7 ± 6.0% (*p* < 0.01) in the OM, and 35.3 ± 2.7% (*p* < 0.01) in the IM on day 8.

These data indicated that the decreased expression of renal AQP2 was accompanied by evident proteinuria in the present experimental model of nephrotic syndrome, in good agreement with a previous report [18].

### 2.5. Excretion of Urinary Exosomal Marker Proteins after PAN Treatment

**Figure 4:**
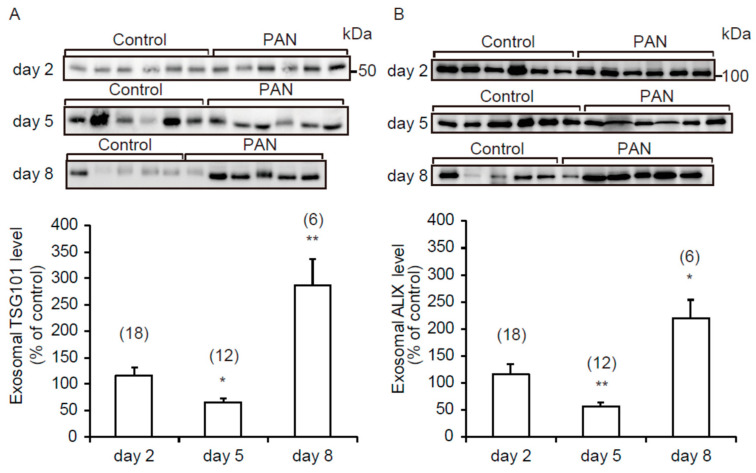
Urinary excretion of exosomal tumor susceptibility gene 101 protein (UE-TSG101) and ALG-2 interacting protein X (UE-ALIX) after PAN treatment. Representative immunoblots with quantitative data for UE-TSG101 (**A**) and UE-ALIX (**B**). Each sample was loaded with the same amount of creatinine (100 µg/lane). Each value was calculated as a percentage of the mean value of the control group. Data are expressed as means ± SE. * *p* < 0.05, and ** *p* < 0.01 vs. control group.

TSG101 and ALIX have been reported to play a pivotal role in the biogenesis of exosomes and to be marker proteins for exosomes [8,20]. Representative immunoblots of urinary exosomal TSG101 (UE-TSG101) and ALIX (UE-ALIX) are shown in Figure 4A,B, respectively. Interestingly, both proteins showed the same excretion pattern, including a significant reduction on day 5 and increases on day 8. These data suggest that the number of exosomes released into the urine was decreased on day 5 and increased on day 8.

## 3. Discussion

The kidney is the main center for fluid volume regulation in the body, and AQP2 is one of the molecular components crucial for this function [11,21]. Therefore, when the kidney is damaged, which is accompanied by fluid imbalance, the renal expression of AQP2 is altered. This alteration may be reflected by changes in the excretion of UE-AQP2. In fact, our group has reported that excretion of UE-AQP2 is altered in experimental models of renal injury, including those induced by renal ischemia/reperfusion, cisplatin, and gentamicin [13,14,15,16,17,18,19,22]. To our knowledge, however, no previously reported study has investigated alterations in the excretion of UE-AQP2 in experimental models of nephrotic syndrome. In the present study, we examined the excretion pattern of UE-AQP2 in a PAN-induced experimental model of nephrosis. Urinalysis demonstrated the successful induction of nephrotic syndrome 5 days or later after PAN treatment, as judged by the appearance of proteinuria. The excretion of UE-AQP2 was decreased on days 2 through 8 in the PAN group relative to the control group. The results of immunohistochemistry showed that the renal expression of AQP2 was increased on day 2 and decreased on days 5 and 8. These data indicate that excretion of UE-AQP2 is decreased in PAN-induced nephrotic syndrome and that the decrease reflects AQP2 renal expression in the proteinuria phase.

On day 2 after PAN treatment, UE-AQP2 was clearly decreased, whereas the renal expression of AQP2 was upregulated. At the same time point, immunoblotting analysis of exosomal marker proteins suggested that the number of exosomes released into urine in the PAN group was unchanged relative to that in the control group. These results suggested that the reduction of UE-AQP2 was not simply dependent on the level of renal AQP2 expression. A simple interpretation would be that renal AQP2 retention occurred through inhibition of UE-AQP2 excretion on day 2 in the PAN group. Previously, Sonoda et al. [22] reported that there was a significant negative correlation between excretion of UE-AQP1 and renal AQP1 abundance in the cortex and OM at an early stage of renal injury (e.g., 30 h) after renal ischemia/reperfusion. Although the mechanism involved has not been clarified, the excretion of urinary exosomes may have a role in regulating the integral membrane protein content of the renal epithelium early in renal injury.

In contrast to the early events, on day 5 after PAN treatment, the excretion of UE-AQP2 was clearly decreased, which was accompanied by decreases in the expression of renal AQP2 and excretion of UE-TSG101 and UE-ALIX. The decreased excretion of UE-AQP2 at this time point was thought to be mediated by decreases in both its renal expression and the number of exosomes released into urine in the PAN group.

On day 8 of PAN-induced renal injury, the excretion of UE-AQP2 and the renal expression of AQP2 were both decreased. On the other hand, the excretion of UE-TSG101 and UE-ALIX was increased. Therefore, it appeared that the decreased excretion of UE-AQP2 could be explained by its renal expression and not by the decrease in the number of exosomes. If so, the decreased renal expression would have predominantly outweighed the increase in the number of exosomes released into the urine. In fact, the reduction of renal AQP2 expression in the PAN group was the greatest on day 8 in the IM, where AQP2 is known to be expressed abundantly. Furthermore, a similar case has been reported for renal AQP1 in a rat model of renal ischemia/reperfusion injury [16].

AQP2 plays a critical role in urine concentration in response to vasopressin [11,21]. AQP2 is expressed in the collecting ducts and provides a pathway for water flux at their apical membranes. When the plasma concentration of vasopressin increases as a result of enhanced secretion from the posterior pituitary, the hormone increases the apical expression of AQP2 through a shuttling mechanism and/or increased abundance in the kidney. A phenomenon known as “vasopressin escape”, whereby collecting ducts do not react with vasopressin, has been reported [23,24]. The escape has been observed in the proteinuria phase of experimental models of nephrotic syndrome, and one mechanism for this escape includes a vasopressin-independent decrease in AQP2 in the collecting ducts [18,19]. In the present study, the excretion of UE-AQP2 was decreased when proteinuria was evident in the PAN group, which was accompanied by a decrease in its renal expression. These data suggest that “vasopressin escape” may occur in our model and that a combination of proteinuria and UE-AQP2 could be a possible biomarker of the phenomenon in nephrotic syndrome.

TSG101 is a component of ESCRT-I, and ALIX is associated with ESCRT proteins [1]. As TSG101 and Alix play important roles in the biogenesis of exosomes, these proteins have been used as markers of urinary exosomes [8,20]. In the present study, we observed that the excretion of UE-TSG101 and UE-ALIX was decreased on day 5 and increased on day 8, suggesting that the number of exosomes released into urine was increased on day 5 and decreased on day 8 [8,20]. In the last decade, a considerable number of in vitro studies have examined the underlying mechanisms responsible for the release of exosomes [25], including hypoxia, endoplasmic reticulum stress, autophagy, and intracellular calcium. However, the number of in vivo studies concerning urinary exosomes has been very limited, and only a few have investigated the regulatory mechanisms of exosome release into urine. It seems that a possible enhancement factor could include urine alkalization [26]. However, in the present study, a consistent urinary slight acidification in response to PAN was observed during the experimental period, and thus urinary pH could not explain the PAN-induced alterations in the release of UE-TSG101 and UE-ALIX. Therefore, currently it is difficult to discuss alterations occurring in the opposite direction for the release of UE-TSG101 and UE-ALIX into urine. Further studies will need to examine the mechanism responsible for the altered release of exosomes from renal epithelial cells into urine after PAN treatment.

“Vasopressin escape” is similar to nephrogenic diabetes insipidus (NDI) in terms of the unresponsiveness of AQP2 to vasopressin. Many studies of pharmacological therapeutic approaches for NDI have already been reported [27]. Such drugs include non-steroidal anti-inflammatory drugs, the diuretic hydrochlorothiazide, the phosphodiesterase type 5 inhibitor sildenafil, G-protein-coupled receptor agonists, the lipid-lowering agents statins, an activator of the adenosine monophosphate kinase metformin, an inhibitor of A-kinase anchoring proteins binding to protein kinase A, activators of the calcium/calmodulin/calcineurin signaling pathway, a P2Y_12_ receptor blocker, and the antimycotic drug fluconazole [27,28,29,30]. Since the present study showed that UE-AQP2 probably mirrors the renal expression of AQP2, UE-AQP2 will be useful for non-invasively detection of vasopressin-independent renal upregulation of AQP2 in response to agents that are expected to be used for “vasopressin escape” and/or NDI.

In summary, we have provided evidence that UE-AQP2 is decreased in PAN-induced nephrotic syndrome. When proteinuria was evident in the PAN group, this decrease reflected the renal expression of AQP2. “Vasopressin escape” is known to occur in nephrosis, possibly due to a decrease in the renal expression of AQP2. Therefore, in terms of urine biochemistry, combined measurement of proteinuria and UE-AQP2 may be used to predict “vasopressin escape” in nephrotic syndrome. For better evaluation of UE-AQP2 in nephrosis, additional human clinical studies will be necessary.

## 4. Materials and Methods

### 4.1. Chemicals and Antibodies

PAN was from Wako Pure Chemical Industries (Osaka, Japan). Rabbit anti-AQP2 polyclonal antibody (catalog no. AQP-002) was from Alomone Labs (Jerusalem, Israel), rabbit anti-TSG101 monoclonal antibody (catalog no. ab125011) was from Abcam (Cambridge, UK), goat anti-ALIX polyclonal antibody (catalog no. sc49268) was from Santa Cruz Biotechnology (Santa Cruz, CA, USA), peroxidase-conjugated anti-rabbit IgG antibody (catalog no. 7074) was from Cell Signaling Technology (Danvers, MA, USA), and anti-goat IgG antibody (catalog no. P0449) was from Dako Japan (Tokyo, Japan), were used.

### 4.2. Animals and Experimental Protocol

A total of 36 male Sprague Dawley (SD) rats aged 9 weeks were purchased from Kyudo (Saga, Japan). The animal studies were conducted in accordance with the Guide for the Care and Use of Laboratory Animals at the University of Miyazaki with a review and approval of the University of Miyazaki Animal Care and Use Committee (approval numbers #2012-009-2-6 in March 2013–2017). Finally, one animal was excluded, due to sampling failure.

All animals had free access to water and standard food. They were divided randomly into two groups: a control and a PAN group that received a single intraperitoneal injection of saline or PAN (125 mg/kg body weight), respectively, on day 0. The experiment was ended on day 8. Blood, urine, and kidneys were collected on days 2, 5, and 8. The blood was obtained at the time of kidney removal. For urine collection, all rats were allocated to individual metabolic cages with free access to tap water.

### 4.3. Measurement of Blood and Urine Parameters

Creatinine, electrolyte (Na^+^, K^+^, and Cl^−^) and blood urea nitrogen concentrations, and blood pH were analyzed using an autoanalyzer (Fuji Film Medical, Tokyo, Japan) or an i-STAT system (Abbott Laboratories, Chicago, IL, USA). Urine pH was measured by a pH meter (ISFTCOM Co., Ltd., Saitama, Japan). Urinary protein concentration was determined using the quick start Bradford protein assay kit (Bio-Rad Laboratories Co., Hercules, CA, USA).

### 4.4. Isolation of Urinary Exosomes

Differential centrifugation was used for the isolation of urinary exosomes, as described previously [27]. Briefly, urine was collected from rats for 6 h. Immediately after collection, the urine was centrifuged at 1000× *g* for 15 min, and the supernatant was centrifuged at 17,000× *g* for 15 min to remove urinary debris. The resulting supernatant was retained, and the pellet was resuspended in an isolation solution (250 mM sucrose, 10 mM triethanolamine, 50 mg/mL DTT) and then incubated at 37 °C for 10 min. Subsequently, the sample was centrifuged again at 17,000× *g* for 15 min. The first and second supernatants were combined, and the combined solution was ultracentrifuged at 200,000× *g* for 1 h (Optima TL Ultracentrifuge; Beckman Instruments, Brea, CA, USA). The resulting pellet was solubilized in a 10-fold diluted protease inhibitor mixture, and then the suspension was mixed with 4× sample buffer (8% SDS, 50% glycerol, 250 mM Tris-HCl, 0.05% bromophenol blue, 200 mM DTT), followed by incubation for 30 min at 37 °C. The sample was stored at −80 °C for further experiments.

### 4.5. Western Blotting Analyses

Urinary exosomal proteins were separated by SDS-PAGE (each sample was loaded in a lane with the same amount of creatinine) [22,31] and then transferred to polyvinylidene difluoride membranes. After blocking with 5% skim milk in 0.05% Tween-Tris-buffered saline (TTBS), the membrane was incubated with 1.5% skim milk in TTBS including a primary antibody (anti-AQP2, -TSG101, or –ALIX antibody) at 30 °C for 1 h. The membrane was then incubated with 1.5% skim milk in TTBS including a secondary antibody at 30 °C for 45 min. Bands were visualized using a Super Signal chemiluminescence detection system (Thermo Fisher Scientific Inc., Waltham, MA, USA) and quantified using the ImageQuant TL software (GE Healthcare, Uppsala, Sweden). Corresponding control samples from animals treated with vehicle were always loaded in each gel for normalized quantification.

### 4.6. Histopathology and Immunohistochemistry

Formalin-fixed kidney samples were embedded in paraffin blocks and cut into 2-μm-thick sections. The sections were stained with the periodic acid–Schiff (PAS) reagent (Muto Pure Chemicals Co., Ltd., Tokyo, Japan) as described previously [16].

For immunohistochemical examination, the paraffin sections were deparaffinized and dehydrated sequentially in a graded ethyl alcohol series. The antigen was then retrieved by autoclaving at 121 °C for 5 min. Next, endogenous peroxidase was inactivated with a 3% H_2_O_2_ solution for 5 min. The slide was then blocked in a solution containing 5% BSA for 20 min. Thereafter, the slide was incubated with anti-AQP2 antibody for 1 h at 37 °C, followed by incubation with Envision System Labelled Polymer reagent (Dako Japan, Tokyo, Japan) for 45 min at 37 °C. The reaction product was visualized by treatment with 3,3′-diaminoben-zidine, and the slide was counterstained with Mayer’s hematoxylin. Each specimen was scanned, and its image was acquired using a NanoZoomer 2.0 RS virtual slide scanner (C10730-13, Hamamatsu Photonics K.K., Shizuoka, Japan) with the NDP.view2 software package (U12388-01, Hamamatsu Photonics K.K.).

Five fields each for cortex, OM, and IM from one animal kidney were analyzed semi-quantitatively using the WinROOF image system (Mitani Co., Tokyo, Japan). The relative AQP2-positive area was calculated by determining the ratio of the AQP-positive area to the total area. The mean value for the control group was considered to be 100%.

### 4.7. Statistical Analysis

All quantitative data are represented as means ± SE. Statistical comparisons between the control and the PAN treated groups were accomplished by Student’s *t*-test using SPSS (Statistical Package for the Social Sciences, version 21.0, Chicago, IL, USA); *p* values < 0.05 were considered to indicate statistical significance.

## Figures and Tables

**Table 1:** Changes in blood parameters after puromycin aminonucleoside (PAN) treatment.

Parameters	Group	Day 2	Day 5	Day 8
**Plasma Creatinine (mg/dL)**	Control	0.18 ± 0.02 (*n* = 6)	0.15 ± 0.02 (*n* = 6)	0.16 ± 0.02 (*n* = 5)
	PAN	0.20 ± 0.00 (*n* = 6)	0.48 ± 0.10 * (*n* = 6)	0.45 ± 0.10 * (*n* = 6)
**Blood pH**	Control	7.35 ± 0.01 (*n* = 6)	7.35 ± 0.01 (*n* = 6)	7.34 ± 0.01 (*n* = 5)
	PAN	7.34 ± 0.01 (*n* = 6)	7.36 ± 0.01 (*n* = 6)	7.39 ± 0.01 ** (*n* = 6)
**Blood Na^+^ (mmol/L)**	Control	140.83 ± 0.31 (*n* = 6)	141.67 ± 0.21 (*n* = 6)	141.00 ± 0.45 (*n* = 5)
	PAN	140.17 ± 0.70 (*n* = 6)	137.00 ± 1.15 ** (*n* = 6)	139.00 ± 0.68 * (*n* = 6)
**Blood K^+^ (mmol/L)**	Control	4.48 ± 0.07 (*n* = 6)	4.67 ± 0.13 (*n* = 6)	4.58 ± 0.12 (*n* = 5)
	PAN	4.43 ± 0.11 (*n* = 6)	5.25 ± 0.19 * (*n* = 6)	4.52 ± 0.14 (*n* = 6)
**Blood Cl^−^ (mmol/L)**	Control	102.00 ± 0.58 (*n* = 6)	101.50 ± 0.22 (*n* = 6)	100.40 ± 0.68 (*n* = 5)
	PAN	101.00 ± 1.06 (*n* = 6)	105.33 ± 0.92 ** (*n* = 6)	101.33 ± 1.28 (*n* = 6)

Data are expressed as means ± SE. * *p* < 0.05, and ** *p* < 0.01 vs. control group.

**Table 2:** Changes in urinary parameters after PAN treatment.

Parameters	Group	Day 2	Day 5	Day 8
**Urinary creatinine (mg/6 h)**	Control	6.37 ± 0.29 (*n* = 18)	5.14 ± 0.43 (*n* = 12)	6.93 ± 0.48 (*n* = 5)
	PAN	6.54 ± 0.59 (*n* = 18)	3.65 ± 0.48 ** (*n* = 12)	4.46 ± 0.77 * (*n* = 6)
**Urine volume (ml/6 h)**	Control	9.28 ± 0.49 (*n* = 18)	9.60 ± 0.89 (*n* = 12)	15.01 ± 1.32 (*n* = 5)
	PAN	9.93 ± 0.97 (*n* = 18)	4.53 ± 0.70 *** (*n* = 12)	8.68 ± 1.21 ** (*n* = 6)
**Urinary protein (mg/6 h)**	Control	4.48 ± 0.29 (*n* = 18)	4.39 ± 0.37 (*n* = 12)	5.56 ± 0.70 (*n* = 5)
	PAN	5.95 ± 0.81 (*n* = 18)	88.46 ± 19.99 *** (*n* = 12)	184.09 ± 62.48 * (*n* = 6)
**Urine pH**	Control	7.23 ± 0.07 (*n* = 18)	7.39 ± 0.06 (*n* = 12)	7.20 ± 0.19 (*n* = 5)
	PAN	6.15 ± 0.11 *** (*n* = 18)	6.44 ± 0.24 *** (*n* = 12)	6.30 ± 0.27 * (*n* = 6)
**Urinary Na^+^ (mEq/6 h)**	Control	0.95 ± 0.07 (*n* = 18)	0.66 ± 0.09 (*n* = 12)	0.89 ± 0.06 (*n* = 5)
	PAN	0.35 ± 0.06 *** (*n* = 18)	0.23 ± 0.09 ** (*n* = 12)	0.53 ± 0.13 * (*n* = 6)
**Urinary K^+^ (mEq/6 h)**	Control	1.00 ± 0.06 (*n* = 18)	0.77 ± 0.06 (*n* = 12)	0.90 ± 0.11 (*n* = 5)
	PAN	0.56 ± 0.06 *** (*n* = 18)	0.33 ± 0.08 *** (*n* = 12)	0.57 ± 0.10 * (*n* = 6)
**Urinary Cl^−^ (mEq/6 h)**	Control	0.80 ± 0.05 (*n* = 18)	0.56 ± 0.07 (*n* = 12)	0.81 ± 0.11 (*n* = 5)
	PAN	0.34 ± 0.05 *** (*n* = 18)	0.17 ± 0.08 *** (*n* = 12)	0.38 ± 0.06 ** (*n* = 6)
**Urinary osmolality** **(mOsm/kg H_2_O)**	Control	828.8 ± 54.8 (*n* = 18)	698.4 ± 103.1 (*n* = 12)	843.0 ± 245.5 (*n* = 5)
	PAN	629.6 ± 66.6 * (*n* = 18)	725.7 ± 58.4 (*n* = 12)	508.5 ± 59.6 (*n* = 6)

Data are expressed as means ± SE. * *p* < 0.05, ** *p* < 0.01, and *** *p* < 0.001 vs. control group.

## Abbreviations

ALIX	ALG-2 interacting protein X
AQP	aquaporin
ESCRT	endosomal sorting complex required for transport
MVB	multivesicular body
PAN	puromycin aminonucleoside
TSG101	tumor susceptibility gene 101 protein
UE	urinary exosome
